# Experimental evaluation of the deadtime phenomenon for GM detector: deadtime dependence on operating voltages

**DOI:** 10.1038/s41598-020-75310-3

**Published:** 2020-11-17

**Authors:** Bader Almutairi, Syed Alam, Tayfun Akyurek, Cameron S. Goodwin, Shoaib Usman

**Affiliations:** 1 Mining and Nuclear Engineering, Missouri University of Science and Technology, Rolla, MO 65401 USA; 2 Department of Physics, Faculty of Art and Science, Marmara University, 34722 Kadikoy, Istanbul Turkey; 3 Present Address: Rhode Island Atomic Energy Commission, 16 Reactor Rd., Narragansett, RI 02882 USA; 4 Environment and Life Sciences Center, Kuwait Institute for Scientific Research, 13109 Kuwait City, Kuwait

**Keywords:** Engineering, Physics

## Abstract

A detailed analysis of Geiger Mueller counter deadtime dependence on operating voltage is presented in the manuscript using four pairs of radiation sources. Based on two-source method, detector deadtime is calculated for a wide range of operating voltages which revealed a peculiar relationship between the operating voltage and the detector deadtime. In the low voltage range, a distinct drop in deadtime was observed where deadtime reached a value as low as a few microseconds (22 µs for ^204^Tl, 26 µs for ^137^Cs, 9 µs for ^22^Na). This sharp drop in the deadtime is possibly due to reduced recombination with increasing voltage. After the lowest point, the deadtime generally increased rapidly to reach a maximum (292 µs for ^204^Tl, 277 µs for ^137^Cs, 258 µs for ^22^Na). This rapid increase in the deadtime is mainly due to the on-set of charge multiplication. After the maximum deadtime values, there was an exponential decrease in the deadtime reaching an asymptotic low where the manufacturer recommended voltage for operation falls. This pattern of deadtime voltage dependence was repeated for all sources tested with the exception of ^54^Mn. Low count rates leading to a negative deadtime suggested poor statistical nature of the data collected for ^54^Mn and the data while being presented here is not used for any inference.

## Introduction

Radiation detector deadtime has been a phenomenon of interest for scientists and engineers for decades. For any detector system, two events must be separated by a minimum time interval for these events to be recorded as independent. This minimum separation time is called the detector deadtime^1–3^. Deadtime depends on the detector’s design, operating conditions, and the pulse processing circuitry^4^. The combined deadtime of a measurement system is the sum of all contributing factors, including the detector’s intrinsic deadtime, pulse shaping time associated with the preamplifier and the amplifier, analog to digital conversion time, and the data sorting time (MCA) and storage time^5^. A detailed description of the various contributors to total deadtime is included in radiation detector deadtime review article by Usman and Patil^5^. Generally speaking, the system’s deadtime can be divided into two parts: (1) the internal losses in the detector itself, (2) count losses in the system circuitry, and pulse processing. In many cases, the deadtime is mostly caused by the associated electronics. However, for the case of a GM counter, the processes within the detector itself are the major contributors to the deadtime.

There are two idealized deadtime models: paralyzing and non-paralyzing. These deadtime models are traditionally used in the industry as well as in academia. According to the paralyzing deadtime model, each radiation event will be followed by an extendable deadtime. Unless the time gap between the two sequential events is greater than the deadtime, the subsequent event will not be recorded. In this case, the true count rate (n) is related to the observed or measured count rate (m) by the following expression:

1 $$m=n\,{e}^{-n\tau }$$

where $m$ is the measured or observed count rate, $n$ is the true count rate, and $\tau$ is deadtime. On the other hand, for the non-paralyzing model, each radiation event will not be followed by an extendable deadtime; instead, it will reset to zero. The true count rate for the non-paralyzable model is expressed as follows:

2 $$m= \frac{n}{(1+n\,\tau)}$$

These simple, yet useful models have been extensively discussed and utilized^1,6^. In 1978, Muller^7,8^ provided a rather simplified and generalized deadtime model. Another hybrid deadtime model was proposed by Albert and Nelson^9^. This hybrid model was further developed by Lee and Gardner^10^. This model uses two independent deadtimes combined in one equation as follows:

3 $$m= \frac{n\,{e}^{-n\,{\tau}_{P}}}{1+n \,\tau_{N}}$$

where τ_P_ is the paralyzable deadtime, and τ_N_ is the non-paralyzable deadtime. Lee and Gardner^10^ were able to find the values of their deadtimes by using the least square fitting of decaying of ^56^Mn source. Hou and Gardner proposed an improved version of these deadtime models^11^ by further dividing the paralyzing and non-paralyzing components into three subcomponents. In 2009, another deadtime hybrid model was proposed by Patil and Usman^12^. The hybrid model is mathematically expressed as follows:

4 $$m= \frac{n\,{e}^{-n\,f\,\tau }}{1+n\,\tau\,(1-f)}$$

where τ is the total deadtime, and it is used with a probability-based paralysis factor, f. The paralysis factor value can be any value between 0 and 1. If the paralysis factor is 0, then the hybrid model reduces to a non-paralyzing model. However, if the paralysis factor is 1, then the hybrid model reduces to a paralyzing model. A graphical technique is proposed with a decaying source data to obtain the parameters needed for the model use^12^.

None of these researchers have investigated deadtime dependence on the operating conditions and how, if any impact aging would have on detector deadtime. Akyurek et al.^4^ provided some preliminary data on deadtime dependence on operating conditions and aging. Literature has also been limited to the relationship between pulse shape and detector deadtime. While various regions of operation for gas-filled detectors are well documented^1,6^, not much is available in the literature, establishing a relationship between detector deadtime and the operating voltage. Likewise, the dependence of pulse shape on the operating voltage of a gas-filled detector is not sufficiently discussed in the literature. Another important area of detector deadtime research missing in the literature is the performance of proportional counter in current mode and the impact of deadtime on observed current. All these areas of research are important for the radiation measurement community and any effort in any of these areas will be welcome by the community.

Any detailed analysis of deadtime dependence on the operating voltage is likely to help the community develop a better understating of the fundamental phenomenon of deadtime and its behavior. Here we present detailed data on detector deadtime dependence on the operating voltage. In this research, all data was collected using four different radioactive sources according to the two-source method. The data collected in the current study shows a clear deadtime dependence on the applied voltage. Three of the radioactive sources showed similar pattern on voltage dependence, hinting to a possible phenomenological basis of this behavior. Nonetheless, the fourth radioactive source showed a different behavior and will be discussed further in the results and discussion section.

## Materials and methods

### Materials

**Figure 1:**
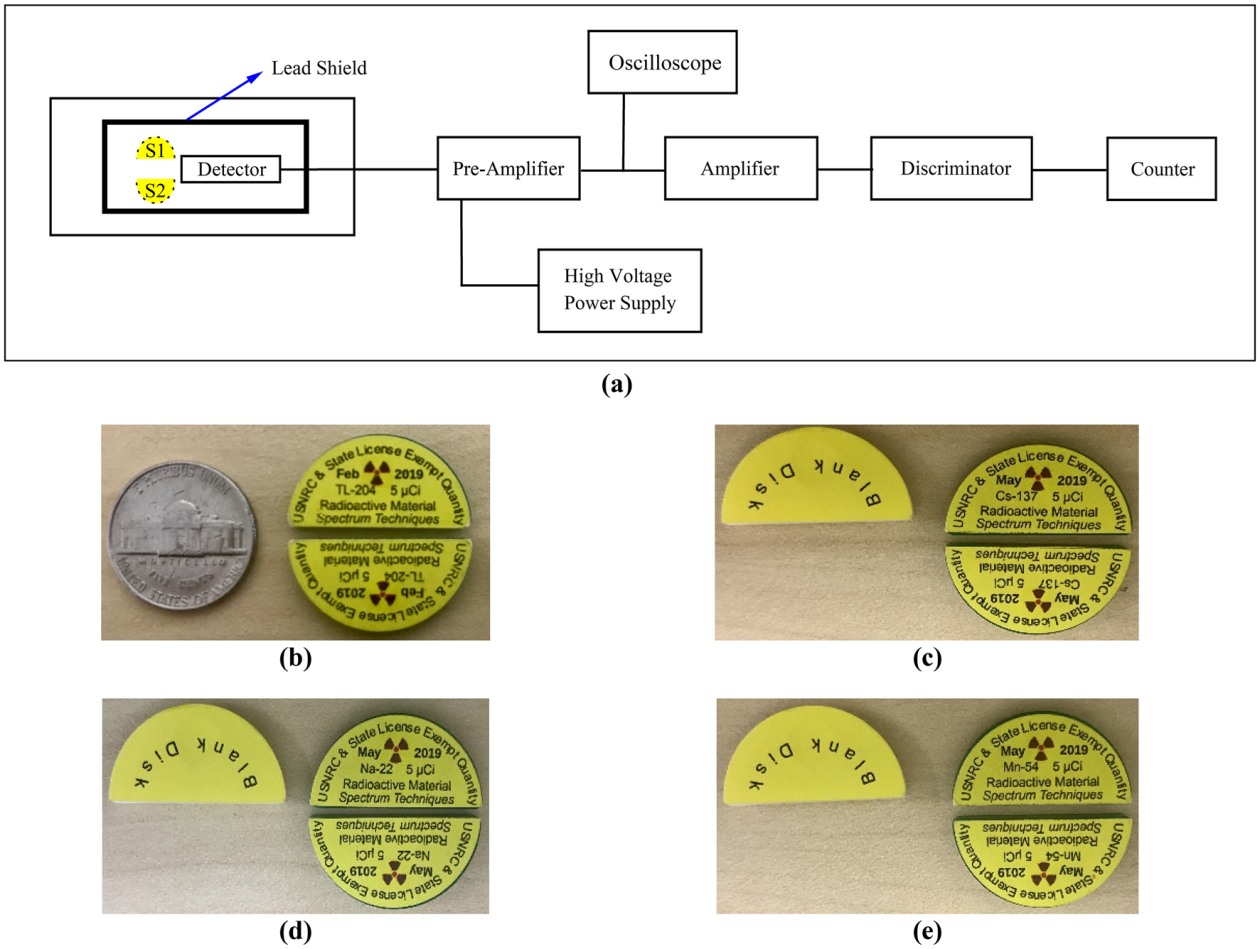
(**a**) Experimental setup for the radiation detection system. (**b**) Thallium-204 split sources used in the experiments. (**c**) Cesium-137 split sources with blank disk. The blank disk filled the position of the second split source when counts were performed to ensure that scattering was unchanged. (**d**) Sodium-22 split sources. (**e**) Manganese-54 split sources.

Figure 1a illustrates the basic experimental setup of the radiation detection system used to evaluate deadtime in this study. The radiation detection system encompasses; a pair of radioactive sources, GM counter, high voltage power supply, pre-amplifier, oscilloscope, amplifier, discriminator, and dual counter/timer.

Radioactive sources of each element consisted of two split sources (the shape of each split source is a half-circle). Four sets of radioactive sources were used in the current study: First, Thallium-204 (^204^Tl)—produced in February 2019. Second, Cesium-137 (^137^Cs), Third, Sodium-22 (^22^Na, and Fourth, Manganese-54 (^54^Mn). The ^137^Cs, ^22^Na, and ^54^Mn sources were all produced in May 2019. Spectrum Techniques specifically produced the ^137^Cs, ^22^Na, and ^54^Mn split sources upon our request for conducting this study. Each split source has an initial activity of 185,000 Bq (5 µCi)^13^. All experiments were conducted in June and July of 2019 during this time the respective source strength were approximately ^204^Tl = 171,310 Bq (4.63 µCi), ^137^Cs = 184,260 Bq (4.98 µCi), ^22^Na = 176,860 Bq (4.78 µCi) and ^54^Mn = 161,690 Bq (4.37 µCi). Nonetheless, the uncertainties of the initial activities of the split sources in this study did not inflict significant measurement or statistical errors. This can be confirmed by the fact that each radioactive split source, when measured individually, resulted in a similar number of registered counts by the GM detector. All sources used in this study had the same size and geometry. Figure 1b–e show the radioactive sources of each element.

A GM detector (Ludlum, model 44-7) was used to detect radiation events. The GM counter is a halogen quenched, end window (a thin mica window) type detector. The counter is able to detect alpha, beta, and gamma radiations. According to the manufacturer of the detector, the typical deadtime of this model is 200 µs at the recommended operating voltage of 900 V. The sensitivity of the detector for ^137^Cs is 2100 cpm/mR/h^14^. A charge-sensitive pre-amplifier (Ortec, model 142A) was used to extract the signals from the detector without degradation of the signal-to-noise ratio. The pre-amplifier was placed as close as possible to the detector to keep the signal degradation at its lowest. The pre-amplifier was connected to the GM detector through a connector series “C” with a coaxial cable^15^. A high voltage (HV) power supply (Canberra, model 3125) was connected directly to the AC line. The HV is capable of providing 0–5000 V bias voltage with 0–300 µA output current. The HV is housed in the nuclear instrumentation module (NIM) along with the amplifier, discriminator, and dual counter/timer. The HV was connected to the input bias of the pre-amplifier through a coaxial cable^16^. An oscilloscope (Tektronix, model TBS2000) was connected directly to the pre-amplifier through a “T” connector^17^. The oscilloscope was used to record all properties of the generated pulses by the GM counter after being processed by the pre-amplifier. The generated pulses recorded by the oscilloscope are discussed in detail in a companion paper^18^. An amplifier (Ortec, model 570) was used to magnify the amplitude of the pre-amplifier output pulse. The amplifier was connected to the pre-amplifier through a “T” connector and to the discriminator through a coaxial cable. The direct-reading gain factor of the amplifier is adjustable from X1 to X1500. In addition, the coarse gain has six-position for selecting the feedback resistors for the gain factor of 20, 50, 100, 200, 500, and 1 K. The amplifier has a shaping time capability that selects the time constant for an active filter network in which the selections are 0.5, 1, 2, 3, 6, and 10 µs^19^. An integral discriminator (Canberra, model 832) was utilized to produce logic output pulses when the linear input pulses amplitude from the amplifier exceeded a threshold. The discriminator level is adjustable from 0 to 10 V^20^. The discriminator is connected to the counter/timer through a coaxial cable. A counter/timer (Ortec, model 994) was used in order to set the timer and display the number of radiation incidents taking place in the GM counter. The counter has a time-base option where time can be specified by a preset value and can range from 0.01 to 990,000 s or 0.01 to 990,000 min^21^.

### Methods

The standard two-source measurement method was utilized for deadtime-voltage dependence measurements throughout this study. This method is based on measuring count rates at an applied voltage over three parts while using: the split sources individually; and a combination of the split sources. Since count losses are nonlinear in this type of experiment, the measured count rates of the combined sources should result in fewer measured counts than if split sources 1 & 2 were summed up individually. For brevity, split source one and split source two are called S1 and S2, respectively, while split sources 1 & 2 combined are called S12.

Previous studies have reported that a GM counter suffers 5% or less paralysis factor; henceforth, the assumption of using a non-paralyzing model while using the simple two-source measurement method for the GM counter is justifiable^4,5,10^. The method’s details are outlined in the Knoll’s textbook^1^. Deadtime was calculated for each applied voltage using the non-paralyzing model based on the following equations:

5 $$X={s}_{1}{s}_{2}-BKG{s}_{12}$$

6 $$Y= {s}_{1}{s}_{2} \cdot \left({s}_{12}+BKG\right)-BKG \cdot {s}_{12} \cdot ({s}_{1}+{s}_{2})$$

7 $$Z=\frac{Y({s}_{1}+{s}_{2}-{s}_{12}-BKG)}{{X}^{2}}$$

8 $$\tau =\frac{X(1-\sqrt{1-Z})}{Y}$$

where ${s}_{1}$, ${s}_{2}$, ${s}_{12}$ are measured count rates of split source 1, split source 2, and split sources 1 & 2 combined, respectively. BKG stands for the background counting rate measurement and $\tau$ stands for deadtime. Since the two-source method depends on observing the difference between large numbers of ${s}_{1}$ and ${s}_{2}$, careful measurements were carried out. A series of experiments were conducted using ^204^Tl split sources at the early stage of this study to optimize each instrument in the detection system. The optimization is based on manipulating the instruments over the GM voltage range region in order to achieve a fractional deadtime of S_12_ of at least 20% and not exceeding 40%. The only instruments manipulated during this optimization effort were the amplifier and the discriminator. Pulse shape setting controlled by the amplifier exhibited the most profound impact. This was performed because the calculated true count rates and deadtimes become sensitive to small variations in measured count rates.

In this section, the details of the experimental method are presented. The split sources were placed on a paper on a tray in a rack (the rack—holds the radioactive sources and GM detector on position inside the lead shield). The position of the split sources was marked on the paper for the subsequent measurements. This step was performed to ensure that the split sources of the various elements during each experiment have the same location and geometry. Hence, the same solid angle applied to all of the duplicated experiments. In order to obtain the optimal results of deadtime, a fractional deadtime $({s}_{12}\, \tau )$ of at least 20% was designed to achieve in the GM operating range^1^. Based on the results of multiple repeated experiments to obtain the desired fractional deadtime, it was determined that the optimal shelf level to hold the tray with the radioactive sources for all experiments was the second from the top of the rack. When the source is placed on the second shelf level, the distance between the source and the detector end-window is 20.65 mm. However, this was not the case with Manganese-54 (Mn-54) because it resulted in fewer observed count rates. Nevertheless, Mn-54 radioactive split sources were also placed on the second shelf in order to be consistent and comparable to the other utilized sources.

To attain the fractional deadtime of 20%, each instrument in the detection system was adjusted. Consequently, the amplifier’s gain was set at 0.5, while the coarse gain at 1 K. Even though the GM detector and radioactive sources were housed inside a lead shield to reduce the background radiation, the discriminator was used to reduce the background noise further. The discriminator level was set at 5 V. The timer was set for 30 min for each experiment. The 30 min duration was sufficient time for counting for our purposes in order not to get negative deadtime values (Mn-54 did produce two negative deadtime and the reasoning behind will be discussed further in the next section). The lowest applied voltage was 570 V for all radioactive sources because it was the operating voltage where the GM counter started to register radiation events. However, the counts were low at 570 V, which resulted in attaining negative deadtimes; thus, data collection started at 600 V. According to the manufacturer of the GM detector, the detector is prone to damage at higher voltages; therefore, 1200 V was the highest applied voltage investigated. This limited was set solely to ensure the safe operation of the GM counter for the subsequent experiments. Due to this high voltage limit of 1200 V, the discharge region was not observed where the deadtime starts to increase rapidly. The observation of discharge region is beyond the scope of this study.

After the radiation detection system was optimized, background radiation events were measured and recorded at each operating voltage from 600 to 1200 V. In order to investigate the deadtime-voltage relationship in detail, the applied voltages were increased incrementally by 50 V for all experiments. For each applied voltage, the timer was set for 30 min. Background radiation incidents were also counted for 30 min at different operating voltages from 600 to 750 V with 10 V increments. These latter measurements were carried out after observing the unusual behavior of deadtime-voltage dependence measurements for the ^204^Tl, ^137^Cs, and ^22^Na sources at the lower applied voltage range.

Counting measurements using the radioactive sources were performed, and the counting rates due to the ^204^Tl split source were recorded. S1 was measured only with the blank disk. The same process was repeated using S2 with the blank disk. S1 and S2 were combined for the final counting measurement. The series of counting measurements (S1, S2, and S12) were repeated for each operating voltage from 600 to 1200 V with 50 V increments, and from 600 to 700 V with 10 V increments. All the data was recorded and entered manually in origin software for further data processing and analysis.

Similar procedures were followed methodically using the ^137^Cs and ^22^Na sources. However, for ^22^Na sources, operating voltages from 650 to 750 V with 10 V increments were taken instead of 600–700 V. The different applied voltages for ^22^Na sources were based on the shift of observed deadtime behavior at these low voltages. For the ^54^Mn sources, the same methodology was followed; however, only measurements from 600 to 1200 V were conducted. This is because there was no noticeable deadtime behavior at lower operating voltages. The reason behind the difference in the applied voltage range for the ^137^Cs, ^22^Na, and ^54^Mn sources is discussed in the next section.

## Results and discussion

### Thallium-204 source

**Figure 2:**
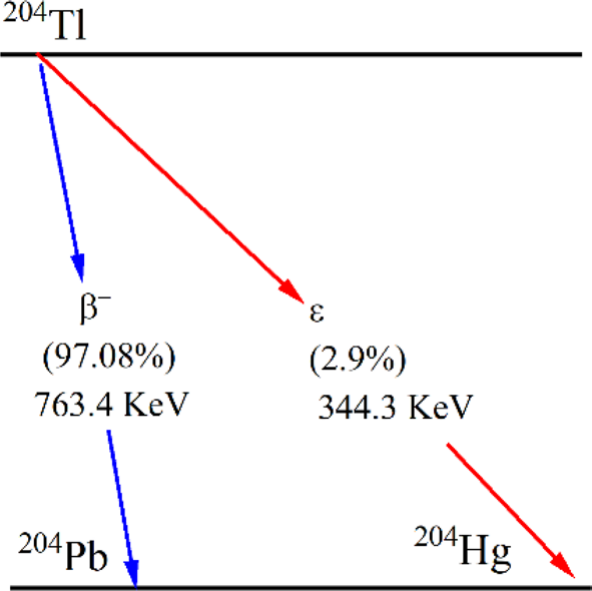
A Schematic of the ^204^Tl decay.

^204^Tl sources were used for the measurement of deadtime at different applied voltages. ^204^Tl decays with a half-life of 3.783 years. The probability mode of decay is 97.08% by beta emission with decay energy of 763.4 keV while 2.92% by electron capture (EC) with 344.3 keV^22^. Figure 2 shows the decay scheme of ^204^Tl.

**Figure 3:**
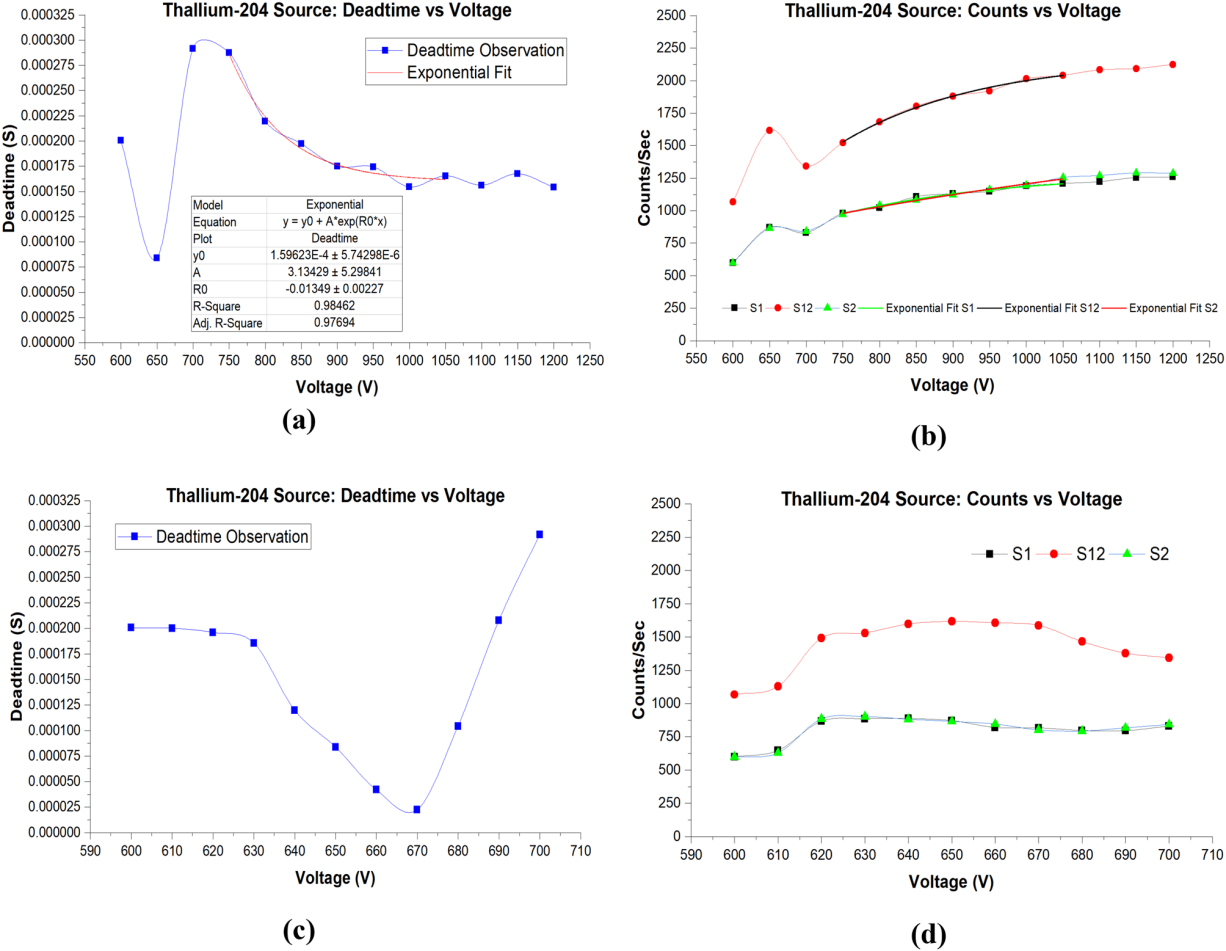
(**a**) Deadtime versus voltage for the ^204^Tl source for the wider range of voltages. Also, the parameters of the exponential fit are shown in the table under the deadtime curve. (**b**) Count rate versus voltage with exponential fits imposed on the curves. (**c**) Deadtime versus voltage for the ^204^Tl source for the narrow voltages range. (**d**) Counts versus voltage for the ^204^Tl for low voltages.

Figure 3a shows voltage versus deadtime from 600 to 1200 V, while the raw data plus the calculated deadtime can be seen in Table 1. At 600 V, deadtime was 200.56 µs. Above the initial voltage, deadtime decreased rapidly to 83.78 µs at 650 V. It was noted that above 650 V, there was a sharp increase in deadtime at 700 V with 291.53 µs. Increasing the voltage further showed a decrease in deadtimes until a plateau was reached from about 1050 to 1200 V.

**Table 1:** Deadtime results at different operating Voltages from 600 to 1200 V.

Voltage (V)	S1 (CPS)	S12 (CPS)	S2 (CPS)	BKG (CPS)	Deadtime (S)
600	598.14 ± 0.57	1067.33 ± 0.77	597.23 ± 0.57	0.14 ± 0.00	2.00E−04 ± 7.88E−07
610	645.02 ± 0.59	1128.46 ± 0.79	627.04 ± 0.59	0.17 ± 0.00	1.99E−04 ± 7.36E−07
620	865.17 ± 0.69	1492.02 ± 0.91	882.30 ± 0.70	0.24 ± 0.01	1.95E−04 ± 5.34E−07
630	884.64 ± 0.70	1530.54 ± 0.92	899.36 ± 0.70	0.29 ± 0.01	1.85E−04 ± 5.07E−07
640	885.14 ± 0.70	1596.81 ± 0.94	880.69 ± 0.69	0.31 ± 0.01	1.19E−04 ± 4.16E−07
650	870.36 ± 0.69	1617.07 ± 0.94	864.50 ± 0.69	0.32 ± 0.01	8.37E−05 ± 3.78E−07
660	818.16 ± 0.67	1606.33 ± 0.94	844.49 ± 0.68	0.31 ± 0.01	4.19E−05 ± 3.51E−07
670	815.44 ± 0.67	1586.81 ± 0.93	800.42 ± 0.66	0.32 ± 0.01	2.24E−05 ± 3.46E−07
680	796.17 ± 0.66	1466.09 ± 0.90	791.46 ± 0.66	0.32 ± 0.01	1.04E−04 ± 4.47E−07
690	793.72 ± 0.66	1378.83 ± 0.87	815.62 ± 0.67	0.32 ± 0.01	2.07E−04 ± 5.95E−07
700	829.44 ± 0.67	1343.46 ± 0.86	841.35 ± 0.68	0.34 ± 0.01	2.91E−04 ± 7.25E−07
750	979.92 ± 0.73	1524.16 ± 0.92	971.86 ± 0.73	0.39 ± 0.01	2.87E−04 ± 6.44E−07
800	1023.18 ± 0.75	1683.34 ± 0.96	1041.96 ± 0.76	0.39 ± 0.01	2.19E−04 ± 5.00E−07
850	1107.85 ± 0.78	1801.61 ± 1.00	1083.49 ± 0.77	0.42 ± 0.01	1.97E−04 ± 4.42E−07
900	1130.41 ± 0.79	1881.12 ± 1.02	1121.53 ± 0.78	0.50 ± 0.01	1.74E−04 ± 3.98E−07
950	1146.96 ± 0.79	1920.77 ± 1.03	1161.72 ± 0.80	2.70 ± 0.03	1.74E−04 ± 3.84E−07
1000	1191.06 ± 0.81	2014.12 ± 1.05	1194.91 ± 0.81	2.02 ± 0.03	1.54E−04 ± 3.46E−07
1050	1208.53 ± 0.81	2041.47 ± 1.06	1254.10 ± 0.83	10.65 ± 0.07	1.65E−04 ± 3.47E−07
1100	1223.97 ± 0.82	2081.71 ± 1.07	1270.07 ± 0.84	13.62 ± 0.08	1.56E−04 ± 3.30E−07
1150	1254.76 ± 0.83	2092.49 ± 1.07	1290.33 ± 0.84	13.99 ± 0.08	1.67E−04 ± 3.41E−07
1200	1258.86 ± 0.83	2123.45 ± 1.08	1287.81 ± 0.84	12.75 ± 0.08	1.53E−04 ± 3.21E−07

S1, S12, S2 are split source 1, split sources 1 & 2 and split Source 2 of Thallium-204, respectively. CPS stands for counts per second whereas BKG stands for background radiation. Deadtime was calculated using the non-paralyzing model.

Furthermore, based on the data collected from 750 to 1050 V, it is observed that there is an exponential decrease. An exponential fit of this range shows a coefficient of determination (R^2^ = 0.98462). These findings reveal a different deadtime relationship behavior than that observed by a previous study conducted by Akyurek et al.^4^ In their study, deadtimes were measured at different operating voltages. But Akyurek et al. only investigated voltages at a narrow range—within 200 V with 10 V increments. According to their study, deadtime showed a linear decrease at low voltages while it plateaued in middle voltages only to linearly increase at higher voltages. Our detailed analysis showed a non-linear behavior suggesting that earlier reported linear behavior could very well be due to limited data points and short range of observation. This deadtime-voltage-dependence linear relationship was not observed in our study. This might be because, in our study, a more extensive range of voltages were investigated with increments of 50 V. It is worth mentioning that the operating voltage was not increased beyond 1200 V because the detector would be damaged; therefore, 1200 V was the highest applied voltage for all the experiments.

Next, deadtime between 600 and 670 V with 10 V increments showed a rapid decrease and followed by a rapid increase from 670 to 700 V. This rapid decrease and increase of deadtime is not reported in the literature. Therefore, we investigated this range more in detail from 600 to 700 V, as can be seen in Fig. 3c. Count rates in this voltage range is shown in Fig. 3d. From 600 to 630, deadtime showed a slight decrease, followed by a rapid decrease from 630 to 670 V, where it showed the lowest calculated deadtime of 22.43 µs. Next, deadtime started to increase to its highest value at 700 V.

Figure 3b shows count rates versus voltage. Split sources 1 & 2 (S12) combined produced more radiation events than if only one split source is used; hence, we will discuss S12 throughout this study. At 600 V, S12 resulted in 1067 counts/s. However, at 650 V, the count rate increased significantly, with a total of 1617 counts/s. This rather high-count rate at a low voltage is mainly due to the fact that observed deadtime was very low, which means that the detector was able to count more radiation events.

Similar to the observed exponential behavior of the deadtime curve between 750 and 1050 V, count rates also showed an exponential behavior; however, it was increasing with increasing applied voltages. The exponential fits can be seen in Fig. 3b, while the parameters for S1, S12, S2 are shown in Table 2. As voltages increased further, count rates increased correspondingly.

**Table 2:** Parameters of the exponential model fit of Fig. 3b using Thallium-204 split sources S1, S12, and S2.

Model	Exponential		
Equation	$y={y}_{0}+A*{e}^{({R}_{0}*x)}$		
Source #	S1	S12	S2
*y*_0_	1288.32 ± 66.78	2170.03 ± 55.72	1905.53 ± 757.71
*A*	− 9035.23 ± 10,164.42	− 34,160.91 ± 21,091.19	− 2142.82 ± 99.87
*R*_0_	− 0.004 ± 0.001	− 0.005 ± 9.16E−4	− 0.0011 ± 0.001
R-square	0.98026	0.99472	0.9914
Adj. R-square	0.97039	0.99208	0.9871

The table was generated through exponential curve fitting analysis using Origin software version (2019b).

The increasing exponential count rate behavior is observed only at higher applied voltages. Nonetheless, the count rate showed different behavior in the narrow range (600–700 V). Count rates increased from 600 to 620 V and plateaued until 670 V, and then it slightly decreased.

### Cesium-137 source

**Figure 4:**
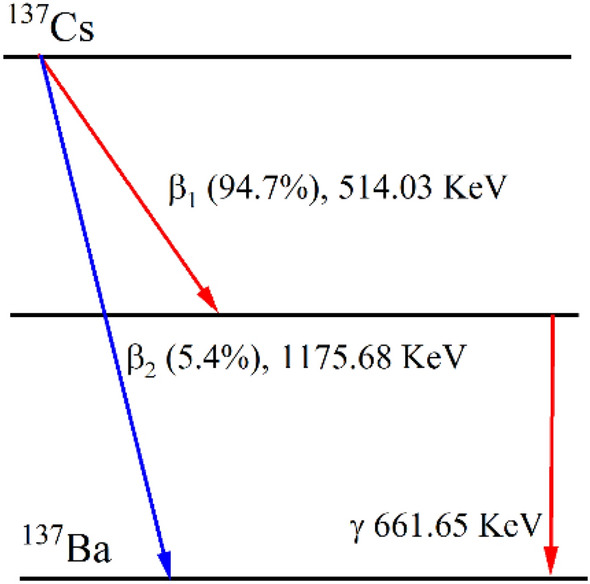
A Schematic of the ^137^Cs decay.

^137^Cs sources were used to measure deadtime at a wide range of operating voltages—600–1200 V. ^137^Cs is produced by nuclear fission in a nuclear reactor. It has a half-life of 30.08 years, and it is both a beta emitter and gamma emitter. The probability mode of decays is the following: (A) about 94.7% by beta emission to ^137m^Ba with decay energy of 514.03 keV. (B) ^137m^Ba has a half-life of 153 s, and it decays into the ground state ^137^Ba about 85.1% with 661.659 KeV^22^. Figure 4 shows the decay scheme of ^137^Cs.

**Figure 5:**
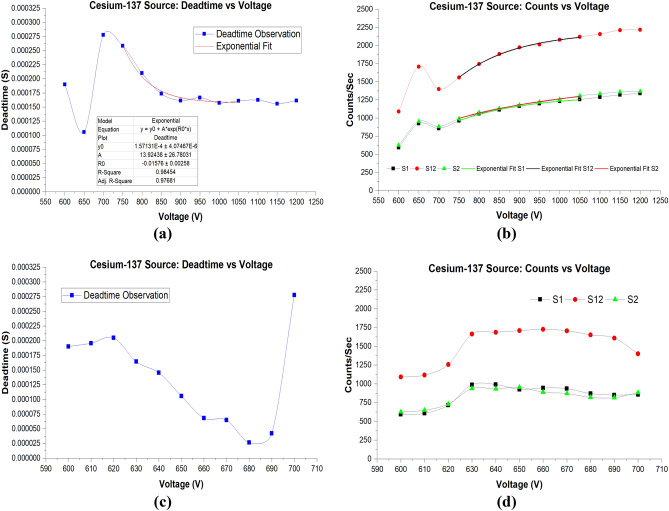
(**a**) Deadtime versus voltage for the ^137^Cs source for the broad range of voltages. Parameters of the exponential fit are shown in the table under the deadtime curve. The parameters were generated using Origin software version (2019b). (**b**) Counts versus voltage with exponential fits imposed on the curves. (**c**) Deadtime versus voltage from 600 to 700 V range. (**d**) Counts versus voltage for the ^137^Cs for the narrow voltages range.

Figure 5a shows the deadtime versus voltage measurements using ^137^Cs sources for the wider range of voltages—600–1200 V. It is clearly seen that deadtime due to ^137^Cs sources revealed similar behavior as measured from ^204^Tl sources, though with different deadtime values. Table 3 shows the split sources and the calculated deadtime for the different applied voltage using ^137^Cs. At 600 V, 650 V, 750 V, deadtimes were 190 µs, 105.45 µs, and 277.49 µs, respectively. Deadtime at 600 V using ^137^Cs sources was 5% higher than the measured deadtime at 600 V using the ^204^Tl sources; on the other hand, at 650 V deadtime using ^137^Cs sources was significantly higher with 20.5%. At 700 V, deadtime using ^137^Cs sources was 5% lower than deadtime using ^204^Tl sources. From 750 to 1050 V, it is observed that the decrease in deadtime revealed a exponential similar to ^204^Tl sources experiments. The exponential fit at this range shows a coefficient of determination (R^2^ = 0.98454). As the applied voltages were increased further, deadtime plateaued.

**Table 3:** Deadtime results for the applied voltages 600–1200 V.

Voltage (V)	S1 (CPS)	S12 (CPS)	S2 (CPS)	BKG (CPS)	Deadtime (S)
600	591.39 ± 0.57	1089.86 ± 0.77	624.35 ± 0.58	0.14 ± 0.00	1.90E−04 ± 7.56E−07
610	605.16 ± 0.57	1115.29 ± 0.78	646.64 ± 0.59	0.17 ± 0.00	1.95E−04 ± 7.42E−07
620	715.18 ± 0.63	1257.70 ± 0.83	728.45 ± 0.63	0.24 ± 0.01	2.04E−04 ± 6.55E−07
630	987.75 ± 0.74	1662.10 ± 0.96	936.74 ± 0.72	0.29 ± 0.01	1.64E−04 ± 4.41E−07
640	990.67 ± 0.74	1686.28 ± 0.96	930.96 ± 0.71	0.31 ± 0.01	1.45E−04 ± 4.14E−07
650	924.31 ± 0.71	1707.70 ± 0.97	952.59 ± 0.72	0.32 ± 0.01	1.05E−04 ± 3.70E−07
660	944.41 ± 0.72	1724.72 ± 0.97	888.21 ± 0.70	0.31 ± 0.01	6.81E−05 ± 3.35E−07
670	935.35 ± 0.72	1705.71 ± 0.97	869.93 ± 0.69	0.32 ± 0.01	6.45E−05 ± 3.38E−07
680	869.93 ± 0.69	1651.37 ± 0.95	818.53 ± 0.67	0.32 ± 0.01	2.64E−05 ± 3.29E−07
690	849.92 ± 0.68	1607.71 ± 0.94	814.24 ± 0.67	0.32 ± 0.01	4.20E−05 ± 3.51E−07
700	855.42 ± 0.68	1399.72 ± 0.88	881.72 ± 0.69	0.34 ± 0.01	2.77E−04 ± 6.79E−07
750	959.95 ± 0.73	1557.57 ± 0.93	989.98 ± 0.74	0.39 ± 0.01	2.58E−04 ± 5.90E−07
800	1058.15 ± 0.76	1743.53 ± 0.98	1076.19 ± 0.77	0.39 ± 0.01	2.09E−04 ± 4.72E−07
850	1112.75 ± 0.78	1880.39 ± 1.02	1134.34 ± 0.79	0.42 ± 0.01	1.73E−04 ± 3.97E−07
900	1165.28 ± 0.80	1971.50 ± 1.04	1179.11 ± 0.80	0.50 ± 0.01	1.61E−04 ± 3.65E−07
950	1199.31 ± 0.81	2012.33 ± 1.05	1219.45 ± 0.82	2.70 ± 0.03	1.66E−04 ± 3.59E−07
1000	1230.46 ± 0.82	2079.25 ± 1.07	1256.23 ± 0.83	2.02 ± 0.03	1.57E−04 ± 3.38E−07
1050	1254.48 ± 0.83	2118.65 ± 1.08	1305.68 ± 0.85	10.65 ± 0.07	1.60E−04 ± 3.30E−07
1100	1288.00 ± 0.84	2156.02 ± 1.09	1333.43 ± 0.86	13.62 ± 0.08	1.62E−04 ± 3.26E−07
1150	1319.07 ± 0.85	2209.92 ± 1.10	1359.38 ± 0.86	13.99 ± 0.08	1.55E−04 ± 3.11E−07
1200	1339.27 ± 0.86	2215.37 ± 1.10	1365.60 ± 0.87	12.75 ± 0.08	1.61E−04 ± 3.17E−07

S1, S12, S2 are source 1, source 1 & 2 and source 2 of Cesium-137, respectively. Deadtime was calculated using the non-paralyzing model.

Next, the differences in deadtime results, specifically at 650 V between ^137^Cs sources and ^204^Tl sources, prompted us to investigate this range in more detail. Figure 5c shows that deadtime from 600 to 620 V increased slightly and then dropped sharply to reach the lowest deadtime of 26.41 µs at 680 V. It is worth noting that the lowest deadtime for ^204^Tl sources was at 670 V, while for the ^137^Cs sources, lowest was at 680 V. This small shift can be attributed to the fact that ^137^Cs is both a beta and gamma emitter. After 680 V, deadtime increased slightly to 42.01 µs at 690 V. Further, deadtime increased significantly at 700 V to reach 277.49 µs, which is the highest observed deadtime for the ^137^Cs experiment. It is also worth noting that deadtimes for 680 and 690 V were below 50 µs unlike with ^204^Tl sources where deadtimes were above 100 µs for the same applied voltages.

Henceforth, it is concluded that there are three distinct deadtime regions in the examined broad range of voltages: (1) Region 1: lower voltages (600–700 V) where deadtime decreases rapidly then increases. (2) Region 2: middle voltages (750–1050 V) where deadtime shows a decreasing exponential behavior. (3) Region 3: higher voltages (1100–1200 V) where deadtime reveals a plateau behavior, as shown in Fig. 5a. On the other hand, count rates showed the opposite behavior to deadtime in region 1 & 2, increasing rather than decreasing with increasing voltages. Nonetheless, count rates in region 3 showed a slight increase rather than a plateau, as indicated in Fig. 5b. Furthermore, the exponential fits and the parameters for S1, S12, S2 in region two are presented in Table 4.

**Table 4:** Parameters of the exponential model fit of Fig. 5b using Cesium-137 S1, S12, S2.

Model	Exponential		
Equation	$y={y}_{0}+A*{e}^{({R}_{0}*x)}$		
Source #	S1	S12	S2
*y*_0_	1316.135 ± 14.99	2191.399 ± 24.16447	1486.62 ± 81.33
*A*	− 24,934.86 ± 8515.02	− 109,809.37 ± 47,646.2833	− 5255.24 ± 2394.57
*R*_0_	− 0.0056 ± 4.99E−4	− 0.00688 ± 6.143E−4	− 0.0031 ± 8.117E−4
R-square	0.99846	0.99787	0.99538
Adj. R-square	0.9977	0.9968	0.99307

The table was generated through exponential curve fitting analysis using Origin software version (2019b).

Figure 5d shows counts versus voltage for region 1. One can notice that there is a slight increase in the number of counts from 600 to 620 V, followed by a significant increase at 630 V and then it plateaued up to 670 V. In the plateau region, a higher number of counts were recorded using ^137^Cs sources compared to ^204^Tl sources. A decrease in observed counts followed the plateau region where the calculated deadtime was at a maximum at 700 V for the ^137^Cs sources.

### Sodium-22 source

**Figure 6:**
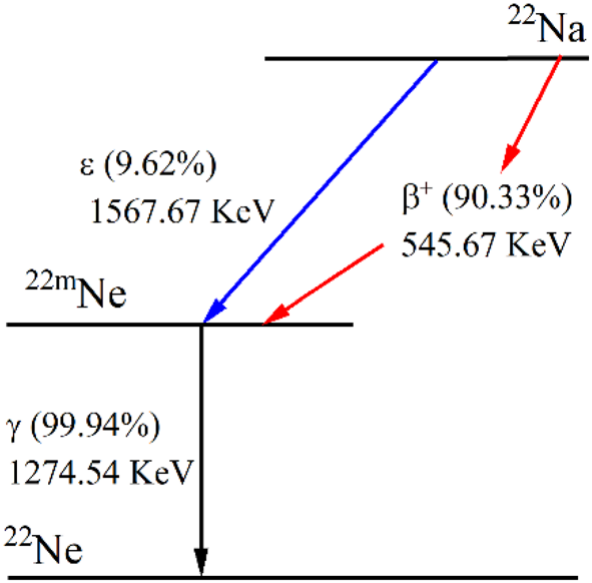
A schematic ^22^Na decay.

^22^Na sources were used to measure deadtime at a wide range of voltages—600–1150 V. Again operating voltages above 1200 V were not investigated to prevent any permanent damage to the GM,hence, 1150 V was the last investigated voltage for ^22^Na. Sodium-22 has a half-life of 2.6018 years, and it is a positron-emitting isotope. ^22^Na is a human-made isotope by the bombardment of aluminum target or high purity magnesium with protons^23^. ^22^Na decays to an excited state of neon with a 9.5% probability via electron capture (EC) with a decay energy of 1567.67 keV. However, it decays mainly via positron emission (90.33%) with decay energy of 545.67 keV, as can be seen in Fig. 6. After only 3.7 picoseconds, the excited neon decays by emitting a 1274.54 keV gamma.

**Figure 7:**
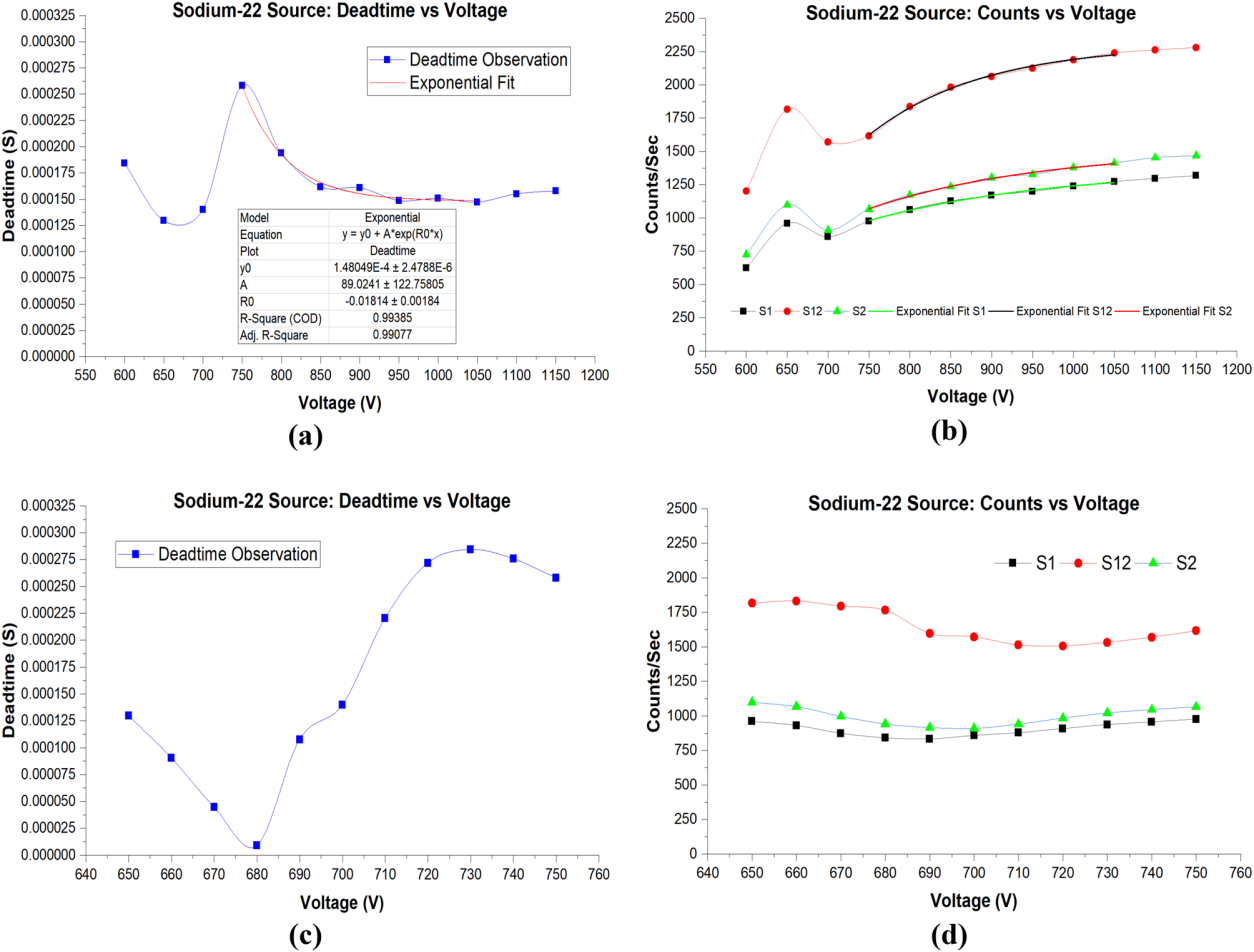
(**a**) Deadtime versus voltage for the ^22^Na source from 600 to 1150 V. Parameters of the exponential fit are shown in the table under the deadtime curve. The parameters were generated using Origin software version (2019b). (**b**) Counts versus voltage with exponential fits imposed on the curves. (**c**) Deadtime versus voltage for the ^22^Na source from 650 to 750 V range. (**d**) Counts versus voltage for the ^22^Na for the narrow voltages range.

Figure 7a shows the deadtime versus voltage measurements using ^22^Na sources for the wider range of voltages of 600–1200 V. Additionally, the exponential model fit parameters are provided under the curve. Table 5 shows CPS for each source and the calculated deadtime at each applied voltage. The deadtime behavior at the lower voltages for ^22^Na showed behavior similar ton ^137^Cs and ^204^Tl sources. However, there was a minor shift in the outcomes at this range. Contrary to deadtime results of ^137^Cs and ^204^Tl where the maximum recorded deadtimes were at 700 V, the maximum calculated deadtime for ^22^Na in the wider range was 257.98 µs at 750 V. The minor shift in the highest calculated deadtime for ^22^Na at 750 V can be attributed to the fact that ^22^Na is a positron-emitting isotope. Since deadtime for ^22^Na at 700 V was also lower than deadtime at 600 V, we selected to investigate the narrow operating voltages from 650 to 750 V instead of 600–700 V.

**Table 5:** Deadtime results at operating voltages from 600 to 1200 V.

Voltage (V)	S1 (CPS)	S12 (CPS)	S2 (CPS)	BKG (CPS)	Deadtime (S)
600	623.48 ± 0.58	1199.82 ± 0.81	724.41 ± 0.63	0.14 ± 0.00	1.84E−04 ± 6.71E−07
650	959.61 ± 0.73	1816.09 ± 1.00	1097.42 ± 0.78	0.32 ± 0.01	1.29E−04 ± 3.67E−07
660	929.47 ± 0.71	1830.89 ± 1.00	1065.83 ± 0.76	0.17 ± 0.00	9.04E−05 ± 3.29E−07
670	872.54 ± 0.69	1793.95 ± 0.99	996.40 ± 0.74	0.24 ± 0.01	4.48E−05 ± 3.04E−07
680	839.82 ± 0.68	1764.89 ± 0.99	939.34 ± 0.72	0.29 ± 0.01	8.94E−06 ± 2.91E−07
690	832.63 ± 0.68	1596.55 ± 0.94	913.802 ± 0.71	0.31 ± 0.01	1.07E−04 ± 4.05E−07
700	857.24 ± 0.69	1571.25 ± 0.93	908.06 ± 0.71	0.34 ± 0.01	1.39E−04 ± 4.44E−07
710	877.32 ± 0.69	1513.23 ± 0.91	938.18 ± 0.72	0.31 ± 0.01	2.20E−04 ± 5.56E−07
720	907.80 ± 0.71	1505.05 ± 0.91	983.32 ± 0.73	0.32 ± 0.01	2.71E−04 ± 6.29E−07
730	935.71 ± 0.72	1530.81 ± 0.92	1019.66 ± 0.75	0.32 ± 0.01	2.84E−04 ± 6.39E−07
740	955.96 ± 0.72	1568.76 ± 0.93	1044.12 ± 0.76	0.32 ± 0.01	2.75E−04 ± 6.13E−07
750	975.47 ± 0.73	1615.62 ± 0.94	1064.31 ± 0.76	0.39 ± 0.01	2.57E−04 ± 5.72E−07
800	1062.51 ± 0.76	1837.12 ± 1.01	1171.41 ± 0.80	0.39 ± 0.01	1.93E−04 ± 4.31E−07
850	1125.76 ± 0.79	1982.75 ± 1.04	1234.43 ± 0.82	0.42 ± 0.01	1.61E−04 ± 3.64E−07
900	1168.58 ± 0.80	2062.00 ± 1.07	1301.54 ± 0.85	0.50 ± 0.01	1.60E−04 ± 3.49E−07
950	1198.30 ± 0.81	2125.34 ± 1.08	1326.16 ± 0.85	2.70 ± 0.03	1.48E−04 ± 3.22E−07
1000	1238.27 ± 0.82	2187.16 ± 1.10	1379.59 ± 0.87	2.02 ± 0.03	1.50E−04 ± 3.16E−07
1050	1272.30 ± 0.84	2237.94 ± 1.11	1411.80 ± 0.88	10.65 ± 0.07	1.47E−04 ± 3.00E−07
1100	1295.83 ± 0.84	2261.37 ± 1.12	1451.83 ± 0.89	13.62 ± 0.08	1.54E−04 ± 3.04E−07
1150	1317.95 ± 0.85	2278.95 ± 1.12	1467.29 ± 0.90	13.99 ± 0.08	1.57E−04 ± 3.05E−07

S1, S12, S2 are source 1, source 1 & 2 and source 2 of Sodium-22, respectively. Deadtime was calculated using the non-paralyzing model.

Furthermore, deadtime at the operating voltages of 750–1050 V followed the same exponential decrease behavior. The exponential fit exhibited a coefficient of determination (R^2^ = 0.99385), as can be seen in Fig. 7b. The R-square of the exponential fit for ^22^Na was higher than that of ^137^Cs and ^204^Tl. This can be explained by the fact that the starting point for the exponential fit was 750 V rather than 700 V. At higher voltages, 1100 and 1150 V, the calculated deadtimes were observed to be steadily increasing rather than showing the plateau behavior comparable to the ^137^Cs and ^204^Tl experiments. However, only two measurements are not sufficient to draw a conclusion to whether an increasing or plateau behavior is to be observed. The missing measurement at 1200 V would have confirmed the progressive increasing or plateauing behavior in this region.

Furthermore, it can be seen from Fig. 7b that the count rates at each applied voltage using ^22^Na were higher than the observed count rates using ^137^Cs and ^204^Tl. The count rate behavior for ^22^Na followed the same pattern as for ^137^Cs and ^204^Tl. Table 6 shows the parameters of the exponential fits for counts at middle voltages for S1, S12, and S2.

**Table 6:** Exponential model fit curve parameters of S1, S12, S2 for Sodium-22 as can be seen from Fig. 7b.

Model	Exponential		
Equation	$y={y}_{0}+A*{e}^{({R}_{0}*x)}$		
Source #	S1	S12	S2
*y*_0_	1363.77 ± 34.86	2302.59 ± 28.09	1515.34 ± 42.30
*A*	− 11,618.90 ± 5675.01	− 150,918.72 ± 76,795.44	− 15,297.80 ± 8330.13
*R*_0_	− 0.0045 ± 7.544E−4	− 0.0072 ± 7.158E−4	− 0.0047 ± 8.326E−4
R-square	0.99626	0.99718	0.99549
Adj. R-square	0.99439	0.99577	0.99323

The table was generated through exponential curve fitting analysis using Origin software version (2019b).

Next, Fig. 7c shows deadtime versus voltage for the narrow range—650–750 V. It is observed that from 650 to 680 V, deadtime is rapidly decreasing. Similar to the ^137^Cs experiment, the lowest deadtime was at 680 V with a deadtime of only 8.94 µs. This is the lowest calculated deadtime obtained from using the radioactive sources in this study. After 680 V, deadtime started to increase up to 730 V where deadtime peaked for the ^22^Na sources at 284.31 µs. It is noteworthy that the lowest calculated deadtimes for ^22^Na compared to ^137^Cs and ^204^Tl were 66.14% and 60.14% lower, respectively, whereas the highest deadtimes were 2.4% higher and 2.53% lower, respectively. It is, therefore, concluded that at lower voltages, the lowest deadtime using ^22^Na is significantly lower than that of the calculated deadtimes using ^137^Cs and ^204^Tl.

Lastly, counts at lower applied voltages for ^22^Na showed different behavior than for ^137^Cs and ^204^Tl, as can be seen in Fig. 7d. From 650 to 680 V, there was slight decrease in count numbers followed by a sharper drop at 690 V. Afterward, there was a steady number of counts from 700 to 750 V.

### Manganese-54 source

**Figure 8:**
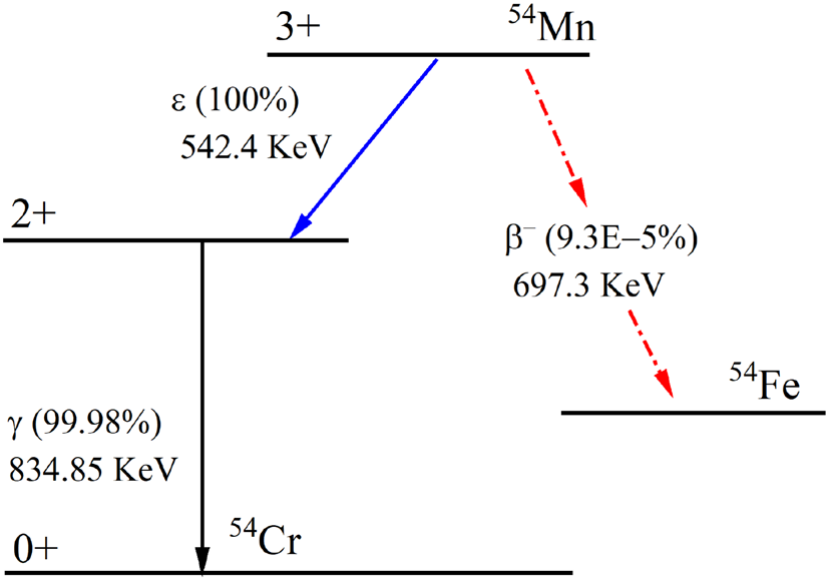
A reduced schematic ^54^Mn decay^25^.

Lastly, ^54^Mn sources were used to compute deadtime from 600 to 1200 V. ^54^Mn has a half-life of 312.2 days. The primary decay mode for ^54^Mn is via EC with a 99.99% probability, followed by photon emission of 834.85 keV. The daughter isotope is a stable ^54^Cr. With a lower probability mode of beta decay (0.000093%), ^54^Mn decays to ^54^Fe, which is also a stable isotope. Nonetheless, with even lower probability mode of decay (5.7E−7%), ^54^Mn decays to ^54^Cr via positron emission^24,25^. Figure 8 shows the reduced decay scheme of ^54^Mn.

**Figure 9:**
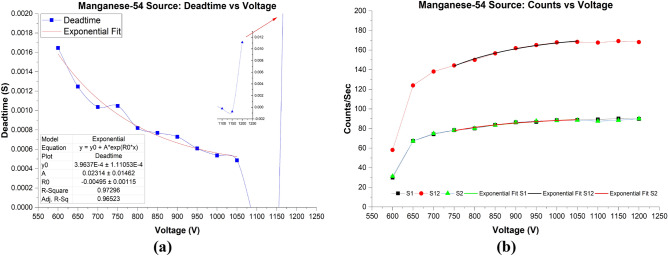
(**a**) Deadtime versus voltage for the ^54^Mn source from 600 to 1200 V. Parameters of the exponential fit are shown under the deadtime curve. The parameters were generated using Origin software version (2019b). (**b**) Counts versus voltage with exponential fits being superimposed on the curves.

Table 7 shows the number of counts of S1, S12, and S2 of ^54^Mn and its calculated deadtimes. It is observed that the number of CPS is significantly lower than the CPS from ^204^Tl, ^137^Cs, and ^22^Na split sources. The lowest CPS of S12 using ^54^Mn sources at 600 V was 94.55%, 94.66%, 95.15% lower than ^204^Tl, ^137^Cs, and ^22^Na, respectively. At the lower voltages, the maximum computed deadtime for ^54^Mn was 1.65 ms at 600 V, followed by an exponential decrease to reach the lowest deadtime of 486 µs at 1050 V. Although the CPS of S12 were steadily increasing at higher voltages, deadtime showed negative values in 1100–1150 V range. In addition, at 1200 V, deadtime had a very high value. The reason for the negative and high deadtimes at these high voltages is due to the high number of observed counts due to background radiation. The BKG showed a rapid increase in the number of counts from 1100–1200 V. When the GM counter registered even higher amounts of BKG events at 1200 V, the calculated deadtime showed significantly higher value with 11.1 microseconds. Figure 9a. illustrates the calculated deadtimes of ^54^Mn radioactive sources as a function of applied voltages.

**Table 7:** Deadtime results at operating voltages from 600 to 1200 V.

Voltage (V)	S1 (CPS)	S12 (CPS)	S2 (CPS)	BKG (CPS)	Deadtime (S)
600	29.96 ± 0.12	58.15 ± 0.17	31.27 ± 0.13	0.17 ± 0.01	1.64E−03 ± 5.07E−05
650	67.39 ± 0.19	123.98 ± 0.26	67.24 ± 0.19	0.32 ± 0.01	1.24E−03 ± 1.77E−05
700	74.21 ± 0.20	138.16 ± 0.27	74.91 ± 0.20	0.36 ± 0.01	1.03E−03 ± 1.48E−05
750	78.29 ± 0.20	144.38 ± 0.28	78.23 ± 0.20	0.39 ± 0.01	1.04E−03 ± 1.40E−05
800	80.09 ± 0.21	150.08 ± 0.28	80.22 ± 0.21	0.46 ± 0.01	8.19E−04 ± 1.26E−05
850	83.63 ± 0.21	156.65 ± 0.29	83.49 ± 0.21	0.48 ± 0.01	7.69E−04 ± 1.18E−05
900	86.33 ± 0.21	161.93 ± 0.29	86.36 ± 0.21	0.68 ± 0.01	7.29E−04 ± 1.11E−05
950	86.75 ± 0.21	165.16 ± 0.30	87.62 ± 0.22	0.54 ± 0.01	6.08E−04 ± 1.06E−05
1000	88.50 ± 0.22	167.92 ± 0.30	88.15 ± 0.22	0.89 ± 0.02	5.36E−04 ± 1.01E−05
1050	88.88 ± 0.22	168.36 ± 0.30	88.58 ± 0.22	2.10 ± 0.03	4.85E−04 ± 9.68E−06
1100	89.22 ± 0.22	167.66 ± 0.30	87.58 ± 0.22	11.82 ± 0.08	− 2.24E−04
1150	90.18 ± 0.22	169.21 ± 0.30	88.33 ± 0.22	17.28 ± 0.09	− 7.38E−04
1200	89.83 ± 0.22	168.22 ± 0.30	90.04 ± 0.22	62.17 ± 0.18	1.11E−02

S1, S12, S2 are source 1, source 1 & 2 and source 2 of Manganese-22, respectively. Deadtime was calculated using the non-paralyzing model.

Next, it is observed from Fig. 9b that the number of counts at low voltages showed different behavior than from the other sources. However, in the middle voltages range, counts demonstrated a similar exponential increase behavior to that of the other sources. Table 8 shows the parameters of the exponential model fits. At 600 V, counts for S12 were very low, followed by a significant increase of counts at 650 V. Then counts followed a steady exponential increase until 1050 V. Counts at higher operating voltages plateaued from 1100 to 1200 V; although, deadtimes were out of characteristics at these higher voltages.

**Table 8:** Exponential model fit parameters of S1, S12, S2 for Manganese-54 Source (Fig. 9b data).

Model	Exponential		
Equation	$y={y}_{0}+A*{e}^{({R}_{0}*x)}$		
Source #	S1	S12	S2
*y*_0_	92.53 ± 2.7589	175.84 ± 3.22	91.49 ± 2.15
*A*	− 588.87 ± 688.42	− 1689.72 ± 1200.67	− 1007.45 ± 1288.91
*R*_0_	− 0.0049 ± 0.001	− 0.005 ± 0.001	− 0.0057 ± 0.001
R-square	0.98023	0.99301	0.979
Adj. R-square	0.97035	0.98951	0.9685

The parameters are generated by curve fitting analysis using Origin software version (2019b).

**Figure 10:**
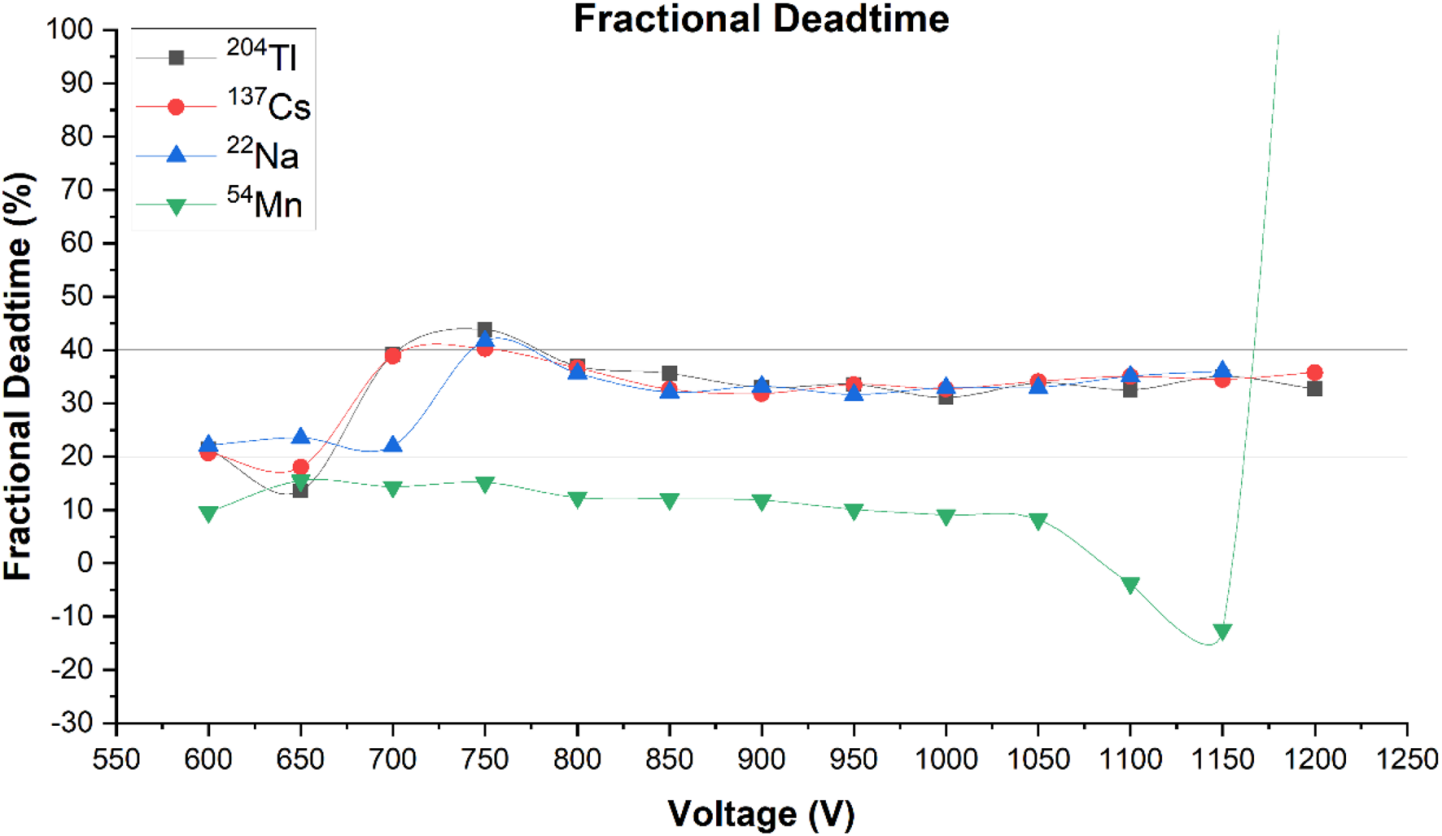
Fractional deadtimes of ^204^Tl, ^137^Cs, ^22^Na, and ^54^Mn sources as a function of applied voltages with reference lines at 20 and 40%.

The experiment utilizing the ^54^Mn sources resulted in negative values for deadtime, at high voltages due to the small number of counts. Deadtime measurements using ^54^Mn sources could be improved by either using a stronger sources of placing the sources on shelf number 1 of the rack instead of shelf number 2. However, for the purpose of evaluating all the sources in similar geometry, the ^54^Mn sources were placed precisely on the second shelf, where other sources were located while performing the experiments. Figure 10 shows a comparison of fractional deadtimes of the sources used in this study. The ^204^Tl, ^137^Cs, and ^22^Na sources from 800 V and above (including GM operating region) are within the acceptable range of fractional deadtime—at least 20% and does not exceed 40%. However, for the ^54^Mn sources, fractional deadtimes were always below 20%, excluding the high fractional deadtime at 1200 V of 187%. Hence, it is determined that the data generated using ^54^Mn sources are not reliable and should not be used to draw further inferences.

## Conclusions

The new data collected and reported is quite revealing. It shows a different behavior of deadtime phenomena, which is not previously discussed in the literature. At low voltage range, a peculiar yet repeatable general deadtime behavior was observed for various radiation sources tested in the experimental research for the GM counting system. After a range of almost constant values, deadtime dropped to a minimum at 650–680 V range before increasing back to a maximum value. The observed peak of deadtime value is observed in the range of 700–750 V. Subsequent to the maximum value, there seems to be an exponential drop in the deadtime, reaching a low asymptotic value during the manufacturer’s suggested operating voltage range of GM counter use (850–1000 V). Furthermore, we also investigated higher operating voltages (1000–1200 V) but did not surpass 1200 V to ensure the safe operation of the GM detector. Therefore, we did not observe the discharge region, where counts increase rapidly. Overall, this behavior of the GM detector’s deadtime has not been reported earlier in the literature.

The observed fall followed by the rapid rise and final exponential fall of deadtime can have a plausible explanation in light of the general behavior of gas-filled detector’s regions of gaseous ionization and charge multiplication.

**Figure 11:**
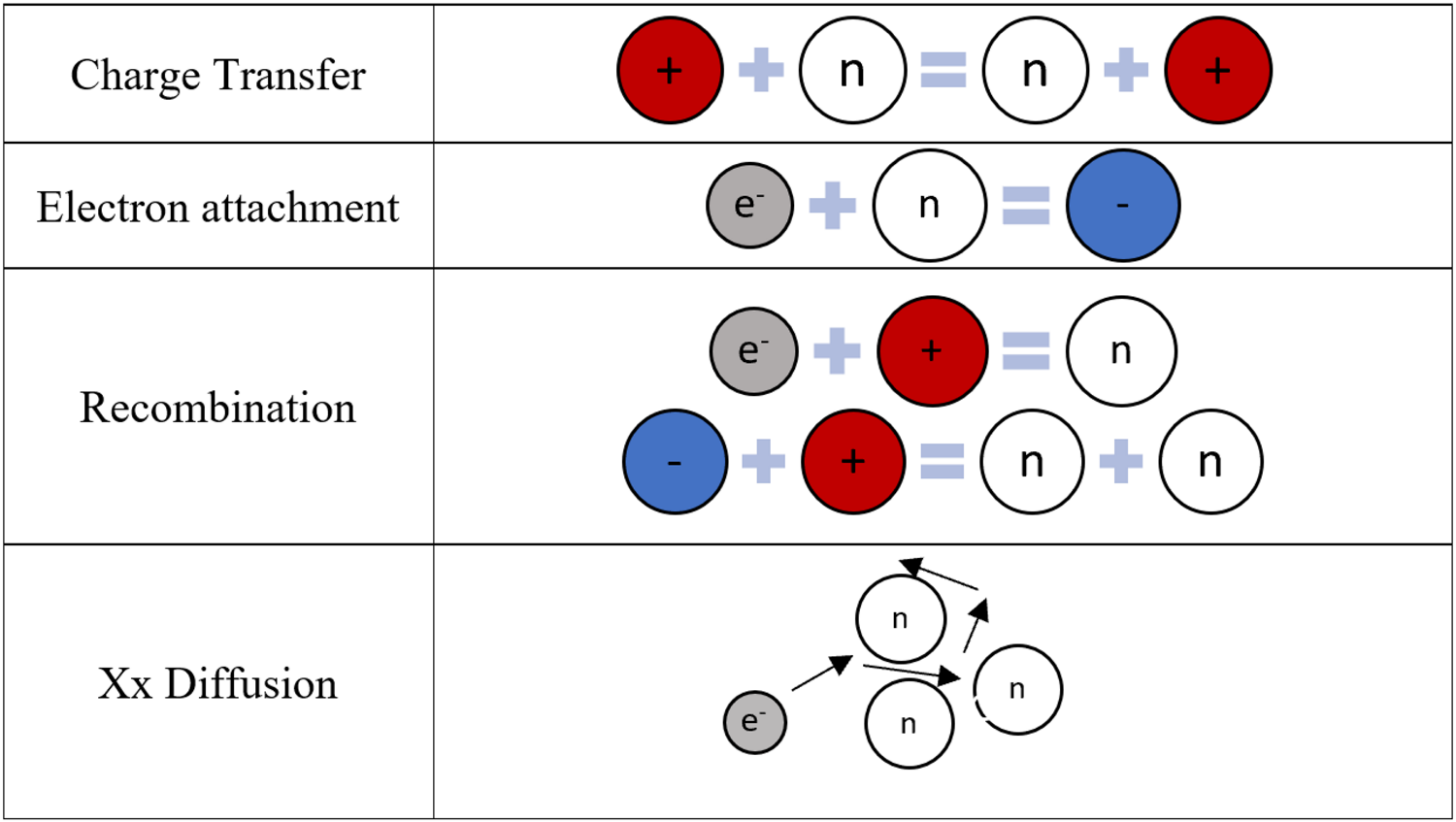
The figure shows different types of interactions of charged species in a gas filled detector. In the left-hand side of the represented equations are the interacting special while the right-hand side are the product of these interactions. The + and – circles represent the positive and negative ions, respectively. The n circles represent a neutral atom or a molecular. The e^−^ circle represents the electrons^26^.

At very low voltage, after initial ionizations take place, free electrons and positive ions drift and diffuse before being collected. At these low voltages, the count rate is low, and not all the events are recorded mainly due to the recombination of positive ions and electrons. Figure 11 shows an illustration of the different types of interactions of charged species. As the voltage increases from these minimal values, there is an observed increase in the count rates and a decrease in the deadtime. The reduced collection time with increasing voltage is the reason for deadtime reduction. A minimum deadtime is reached at the point when the collection time is minimum without any significant charge multiplication. Increasing the voltage further results in charge multiplication, which is caused by the high drift velocity and consequently impacts the energy of electrons. Since each generated pulse is the sum of a larger number of charge carriers, the collection time increases; therefore, deadtime increases. This positive relationship between the operating voltage and deadtime is observed due to the loss of proportionality. Due to the loss of proportionality, no further increase in deadtime is possible. After the maximum is reached, deadtime starts to decrease with increasing the applied voltages with an exponential behavior. Increasing the voltage further results in no significant additional charge multiplication. On the other hand, increasing the voltage reduces the collection time; therefore, deadtime is reduced. The well-known exponential behavior is observed in this range. At 900 V, which is the recommended operating voltage of the detector, a low asymptotic value of deadtime is observed.

This level of detailed GM deadtime analysis has not been reported in the literature. It is hoped that this work will further enhance the radiation measurement’s community understanding of the phenomenon and remedial strategy for dealing with detector deadtime problems.
